# Elemental minerals and microbial compositions as well as knowledge and perceptions regarding kaolin (clay) consumption by pregnant women in the Ho municipality of Ghana

**DOI:** 10.11604/pamj.2019.34.113.17394

**Published:** 2019-10-28

**Authors:** Nii Korley Kortei, Isaac Agyei Annor, George Aboagye, Nana Yaw Barimah Manaphraim, Alice Koryo-Dabrah, Emelia Awude, Edward Ken Essuman, Huseini Wiisibie Alidu, Clement Okraku Tettey, Benedict Awadzi

**Affiliations:** 1 Department of Nutrition and Dietetics, School of Allied Health Sciences, University of Health and Allied Sciences, PMB 31, Ho, Ghana; 2 Department of Medical Laboratory Sciences, School of Allied Health Sciences, University of Health and Allied Sciences, PMB 31, Ho, Ghana; 3 Department of Nutrition and Food Science, School of Biological Sciences, College of Basic and Applied Sciences, University of Ghana, P.O. Box LG 25, Accra, Ghana; 4 Department of Biomedical Sciences, School of Basic and Biomedical Sciences, University of Health and Allied Sciences, PMB 31, Ho, Ghana; 5 Microbiology Unit, Department of Medical Laboratory, Volta Regional Hospital, Ho, Ghana

**Keywords:** Pica, geophagy, knowledge, perceptions, microbial load, minerals

## Abstract

**Introduction:**

Kaolin is a type of clay consumed mostly by women especially pregnant women of which the act of clay eating is termed geophagy. Different people use this type of clay for diverse purposes. Notwithstanding, most Ghanaians consume this clay out of cravings, taste and smell. There have been some attendant problems with the consumption of clay especially by pregnant women. This research sought to assess the mineral and microbial contents of kaolin and address the perceptions of pregnant women on geophagy.

**Methods:**

This study employed a cross-sectional convenient sampling method to sample 217 pregnant women and sellers of clay (Ayilo). A face-to-face interview was conducted to administer a structured questionnaire to respondents. Mineral and microbial analyses were also conducted on the ore of the kaolin mined from Anfoega in the Volta Region of Ghana using standardized procedures.

**Results:**

Results from this study suggests geophagy prevalence of 48.4% (n=217) among pregnant women in the Ho municipality of Ghana. Results obtained also suggest smell and taste of the clay, influenced the consumption by these pregnant women to a large extent. Strikingly, majority of the respondents had no knowledge on the adverse health implications clay consumption had on the human body. The physiological state of pregnancy also cause many to use it to treat nausea, other accompanying discomfort and in some instances to quench their hunger. Traces of Lead, Nickel and Arsenic were found in the clay. Common microorganisms identified were *Bacillus, Pseudomonas, Mucor* and *Aspergillus spp.*

**Conclusion:**

Geophagy is a common practice among pregnant women living in the Ho municipality in the Volta region, Ghana. Most of them consumed it for varied reasons. Although there are beneficial minerals, accumulated effects of these heavy metals can lead to various complications in pregnancy. The clay also contained pathogenic microorganisms. These pathogens have a whole range of deleterious effects on the human body ranging from gastrointestinal infections to cancer and so may not be safe to consume clay products from Anfoega, Ghana.

## Introduction

Pica is a general term which refers to the repeated ingestion of non-food items normally insatiable and leads to its compulsive consumption [1]. Pica in humans has many different subgroups and each of these subgroups is defined by the substance ingested. Based on the items consumed [2], identified thirty-six (36) types of pica which consist of both non-food and food items. Several forms of pica exist and some classifications include amylophagia (starch), coprophagia (faeces), geophagia (dirt, soil and clay), hyalophagia (glass), lithophagia (stones), pagophagia (ice), etc. Furthermore, pica according to other researchers [2-4] include paint, hair, plaster, live wasps, chalk, vinegar, grass, and many others. Geophagy is a form of pica characterized by craving and eating of soil [5]. It is characterized as a form of pica (a term that comes from the Latin word magpie which has indiscriminate eating habits) [6]. The act of eating clay is common in developing countries because most pregnant women eat clay as an appetite suppressant and it is also observed in anorexia nervosa [6]. In Ghana, processed kaolin (white clay) ready for consumption is known as Ayilo (Ga), Agatawe (Ewes), Hyire (Akan) and several other names in other dialects. Kaolin can be industrially mined; it is usually dug out from the deep parts of the earth crust. It is mostly mined in parts of the Volta Region, especially Anfoega, where the freshly mined clayey soil is molded into lumps, oven-baked and distributed to markets across Ghana. It contains chemical elements such as Aluminium, Arsenic, Boron and Nickel which can be potentially harmful to humans [7]. Several scientists have warned against the consumption of clay neither by pregnant women nor ordinary persons, since there are diverse health implications associated with its consumption [8].

The craving for clay, is especially common in pregnant women and it is consumed by different people for different reasons. Most people consume it for its peculiar taste and smell and become addicted to it later on thus preventing them from avoiding it if need be. Kaolin is also perceived to possess some health benefits, it is believed to contain certain mineral elements which could be beneficial to human health, and some spiritual connotations are linked to its intake. They eat the clay because of some traditional beliefs. Some of these beliefs are; an antidote to diarrhoea, stomach discomfort, nausea and other discomforts associated with pregnancy [9]. Geophagy has some economic implications on some families since pregnant women save some monies for its purchase which may be disadvantageous to people with low income. An average of 230 grams of baked clay cost GHS 40 ($9.00) and the price vary with location [10] in Ghana. The objective of this paper therefore was to assess the knowledge, perceptions and mineral contents as well as microbiological quality of clay consumed by women especially pregnant women in Ghana.

## Methods

**Figure 1:**
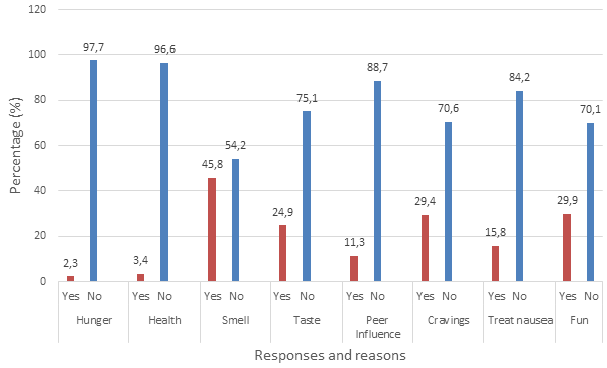
Some reasons why pregnant women practice geophagy

**Study area:** the study area where this research was concluded was in Ho, the capital city of Volta Region (Figure 1). Volta Region is one of Ghana's ten administrative regions. The Kaolin samples were purchased from Anfoega in the Volta Region. Anfoega is situated in Dayi, Volta, Ghana. Its geographical coordinates are 6° 53' 0" North, 0° 18' 0" East and its original name (with diacritics) is Anfoega Akukome [11]. Other samples were taken from the Ho Asigame market.

**Study design:** this study was a cross sectional and experimental study in which both qualitative and quantitative data collection techniques were employed. Questionnaires for data collection were pre-tested by face-to-face interviews and the mineral and microbial contents analyses were done in the laboratory using standard methods. The perception of pregnant women on geophagy was also documented.

**Study population and site:** the study population included individuals who attended antenatal clinic at the Volta Regional and the Ho Municipal Hospitals. The sample size consisted of only pregnant women who had fair knowledge about geophagy. People between age 16 to 45 years were qualified to take part in this study source [12].

**Sample size and sampling procedure:** participants were chosen using convenient sampling and their willingness to take part in the studies. Consent forms were given, it was well explained and those who showed interest in the study were included. Estimated sample size:

$$n=\frac{Z^{2}P\left(1-P\right)}{D^{2}}$$

Where, n = estimated sample size Z = 1.96 (critical value) D = margin of error P = percentage picking a choice (50%) n = (1.96 ²*0.5(1-0.5))/0.05² = (3.8416 *0.5 (0.5))/0.05² = 384.16 Sample size (SS) = 384.16 ÷ (1+ (384.16-1))/(500) = 384.16 ÷ (1+ (384.16-1))/500 = 384.16 ÷ 1.76612 = 217 = 217 participants 217 participants were chosen for the study.

**Questionnaire:** a structured questionnaire was used in taking the data. The questionnaire consisted of demographics, occupation, household background, nutritional knowledge and their perception about geophagy of the respondents.

**Pre-testing of questionnaire:** pretesting was conducted at the Volta Regional Hospital with pregnant women on validity, reliability and accuracy of the questionnaire. The responses proved very helpful as some adjustments were made on the questionnaire. Adjustments ranged from the correction of typographical errors to questions that were difficult to comprehend.

**Data analysis:** data taken was coded and entered with EpiInfo Version 4.2.0.0. The data was then transported into IBM SPSS Statistics 25 for analysis. Univariate analysis was performed for frequencies, means and proportions of sociodemographic characteristics of respondents. Chi-square test was used for the evaluation of significance of difference in association between socio-demographic characteristics and awareness of side-effects. The graphs were drawn with Excel, Microsoft Office 2016. A p-value of <0.001 was considered to be significant.

**Ethical Clearance:** approval was obtained from the Research and Ethics Committee of the University of Health and Allied Sciences, Ho, Ghana. Informed consent of the participants in the study was obtained and respondents were assured of confidentiality of information supplied.

**Sampling of kaolin:** kaolin samples and its final products were bought from Anfoega where the ore was taken and the Ho Asigame Market where final clay samples were purchased for analyses. They were packaged in plastic containers and kept in an ice chest at 4°C and transported under aseptic conditions to the laboratory.

**Sample preparation for mineral analysis:** the clay ore samples were ground into powder with mortar and pestle and sieved using a 0.1 mm mesh. About 100 grams of the samples were weighed and added to 100 ml of distilled water. The samples were then placed on a shaker at 125 rpm for 12 hrs and then allowed to settle. The samples were filtered using a whatman 40 filter paper. The supernatant was then used to run the analysis.

**Determination of mineral elements:** the dry ashing method was used for atomic absorption spectrophotometer (AAS) analysis [13]. All glasswares were washed with 1% nitric acid followed by demineralised water. Three millilitres (3 ml) each of the clay supernatants were weighed into platinum crucibles. The crucible and the test portion were placed in a Muffle furnace at a temperature of 550°C for 8 hrs. The crucible with ash was put in a desiccator to cool. Five millilitres (5 ml) of nitric acid of mass fraction not less than 65%, having a density of approximately ρ (HNO_3_) = 1400 mgml^-1^ was added, ensuring that all the ash came in contact with the acid and the resultant solution heated on hot plate until the ash dissolved. Ten millilitres (10 ml) of 0.1 mol.L^-1^ nitric acid was added and filtered into 50 ml volumetric flask. The resultant solution was topped up to the mark with 0.1 mol.L^-1^ nitric acid. Blank solution was treated the same way as the sample. Buck Scientific 210VGP Flame AAS (Buck Scientific, Inc. East Norwalk, USA) was used to read the absorbance values at appropriate wavelength of the interested metal in the sample solution. Cathode lamps used were Copper (Cu) (wavelength 324.8 nm, lamp current 1.5 mA), Iron (Fe) (wavelength 248.3 nm, lamp current 7.0 mA), Manganese (Mn) (wavelength 279.5 nm, lamp current 3.0 mA), Lead (Pb) (wavelength 217.0 nm, lamp current 3.0 mA) and Zinc (Zn) (wavelength 213.9 nm, lamp current 2.0 mA). Air/acetylene gas was used for all the analyses. The metal content of the samples were derived from calibration curves made up of minimum of three standards.

### Sample preparation for microbiological analysis

**Enumeration of bacteria:** the bacterial analysis was modified from [14]. One gram of each sample was serially diluted in 9 ml sterile distilled water and the resulting dilutions were plated on Blood Agar (BA; CM0271), Sabouraud Dextrose Agar (SDA; Oxoid CM 032) and Potato Dextrose Agar (PDA; Oxoid CM 0325) at 37°C for 48 hrs. The media plates were inspected daily over the 48-hour period. Colonies of resident microorganisms were calculated as CFU/g for each sample. The mean counts of colonies on duplicate plates were determined and colonies were confirmed using appropriate confirmatory tests for the microorganisms present.

**Enumeration of fungi:** this was carried out according to the procedure outlined by [15] with media and process modifications. One (1) gram of each test sample was added separately to 99ml of 0.1% peptone in a 250 ml Erlenmeyer flask and allowed to settle for 5 minutes. Each flask was then shaken at 120 rpm for 10 minutes on an Orbital Shaker (Gallenkamp, England). The samples were serially diluted up to 1:10^4^ and then plated on PDA and SDA followed by incubation at 3°C for 5 days. Moulds and yeast that appeared were identified by their culture and morphological characteristics using standard identification manuals [16].

**Enumeration of parasites:** method described by [17] with modifications. This was done by pouring formol-saline in 15 ml test tube. Approximately 1 g of granulated kaolin was added to the formol-saline and mixed gently until dissolution. The suspension was left standing for about 10 minutes. The suspension was strained through gauze (350-450 nm). Three (3) ml of ether was added to the filtrate and vigorously shaken for 1 minute to obtain a homogeneous mixture. The mixture was then centrifuged for 2 minutes at 2000 rpm. The supernatant was aspirated and discarded. A wet mount was prepared using the sediment and mounted for microscopy.

## Results

**Socio-demographic characteristics of respondents:** a total of 217 pregnant women between the ages of 16-45 years were interviewed in this study. From the social demographics of the study (Table 1), 7.4% of the respondents were aged between 16-19 years, while the rest were between the ages of 20-45 years. Majority 197 (90.8%) of the respondents were Ewes, while the minority 8 (3.7%) were Akans, 8 (3.7%) were Ga-Adangbes and 4 (1.8%) were Northerners. Most of the pregnant women were Christians 197 (90.8%) with the rest being Muslims and Traditionalist. Majority 148 (68.2%) of the pregnant women were married, 60 (27.6%) were single, 4 (1.8%) were widowed and 5 (2.3%) were co-habiting. About 49 (22.6%) of the respondents were self-employed, 60 (27.6%) of the respondents were professional workers, 20 (9.2%) were office workers, 36 (16.6%) were traders and 8 (3.7%) were farmers. Sixteen (7.4%) of them were students and 28 (12.9%) were unemployed. Eighty-four (38.7%) of the pregnant women had no children, 121 (55.7%) of the pregnant women had 1 to 2 children. Twelve (5.5%) of the pregnant mothers had 3 children. One hundred and fifty-six (71.9%) of the respondents had less than 6 household size and 61 (28.1%) had more or equal to 6 household sizes. Greater proportion, 69 (31.8%) of the pregnant women had a total monthly income between GHS 500-800 with just 16 (7.4%) earning between GHS 2000 and 1000 per month. Most 97 (44.7%) of the pregnant women had at least a JHS/Middle School education and 72 (33.2%) had SHS education. Minority, 12 (5.5%) had basic education, 32 (14.7%) had tertiary education qualification and only 4 (1.8%) had no education at all. About 173 (79.7%) of the respondents lived in urban areas.

**Table 1:** Socio-demographic characteristics of respondents

Variables		Frequency	Percentages (%)
**Age of respondents (n=217)**			
	16-19	16	7.4
	20-24	45	20.7
	25-29	64	29.5
	30-34	39	18.0
	35-39	39	18.0
	40-44	14	6.5
**Ethnicity (n=217)**			
	Ewe	197	90.8
	Akan	8	3.7
	Ga-Adangbe	8	3.7
	Northerner	4	1.8
**Religion (n=217)**			
	Christian	197	90.8
	Muslim	16	7.4
	Traditional	4	1.8
**Marital Status (n=217)**			
	Single	60	27.6
	Married	148	68.2
	Divorced	0	0
	Widowed	4	1.8
	Co- Habit	5	2.3
**Occupation (n=217)**			
	Self Employed (carpenter, hairdresser, seamtress)	49	22.6
	Professional (teacher, nurse, nutritionist, lawyer, accountant)	60	27.6
	Office Worker ( secretary, clerk)	20	9.2
	Trading	36	16.6
	Farmer	8	3.7
	Student	16	7.4
	Unemployed	28	12.9
**Number of Children (n=217)**			
	0	84	38.7
	1	61	28.1
	2	60	27.6
	3	12	5.5
**Household size (n=217)**			
	≤5	156	71.9
	≥6	61	28.1
**Total monthly income (n=217)**			
	1000-1999	16	7.4
	800-999	48	22.1
	500-799	69	31.8
	100-499	60	27.6
	<100	24	11.1
**Education level (n=217)**			
	None	4	1.8
	Primary	12	5.5
	Middle/JHS	97	44.7
	SHS/O’level	72	33.2
	Tertiary	32	14.7
**Locality (n=217)**			
	Urban	173	79.7
	Rural	44	20.3

**Pregnant women geophagy practice:** Table 2 summarizes the results obtained from pregnant women who practiced geophagy. Most of the respondents, 108 (49.8%) of 217 had heard of clay (ayilo) from peers, 101 (46.5%) from family and only 8 (3.7%) from the media. Most of them 177 (81.6%) had consumed clay before. Among those who had consumed clay before, quite a moderate number 132 (74.6%) of them consumed clay once during the day. Most of them 116 (65.5%) consumed about 1 piece a day and 12 (6.8%) ate about 4 pieces a day. Those who ate it at any time they felt like were 108 (61%). Most of them, 137 (77.4%) purchased it from the stores and the rest from the market or other places. About 89 (50.3%) of them started eating clay 10-15 years ago, 20 (11.3%) of them 3 months ago and 105 (59.3%) are still eating. Those respondents who had craving to eat clay during pregnancy were 117 (53.9%) and 105 (89.7%) of the 117 eat clay during pregnancy.

**Table 2:** Geophagy practices among pregnant women

Respondents knowledge about	Variables	Frequency	Percentages (%)
Clay (ayilo) (n=217)	Yes	217	100.0
	No	0	0
Where did you hear of Ayilo (n=217)	Media	8	3.7
	Peers	108	49.8
	Family	101	46.5
Have you eaten clay before (n=217)	Yes	177	81.6
	No	40	18.4
How many times do you eat clay in a day? (n=177)	Once	132	74.6
	Twice	25	14.1
	Thrice	8	4.5
	4 or more	12	6.8
Pieces of clay (Ayilo) eaten in a day. (n=177)	1	116	65.5
	2	45	25.4
	3	4	2.3
	4	12	6.8
What time of the day do you usually eat clay? (n=177)	Mornings	12	6.8
	Afternoons	57	32.2
	Evenings	0	0
	Anytime	108	61.0
Where do you purchase your clay from? (n=177)	Stores	137	77.4
	Market	24	13.6
	Others	16	9.0
How long have you been eating clay? (n=177 per each alternative)	15 years	45	25.4
	10 years	44	24.9
	5 years	20	11.3
	3 years	20	11.3
	1 year	16	9.0
	6 months	8	4.5
	3 months	20	11.3
	1 month	4	2.3
	Still consuming clay	105	59.3
Have you had the cravings to eat clay during pregnancy? (n=217)	Yes	117	53.9
	No	100	46.1
Did you eat clay when pregnant? (n=117)	Yes	105	89.7
	No	12	10.26

**Reasons for geophagy practice by pregnant women:** majority 81 (45.8%) of 177 pregnant women consume clay because of the smell (Figure 1). A moderate number of 44 (24.9%) consumed clay because of the taste. Hunger was the reason for 4 (2.3%) and 6 (3.4%) consume clay for health reason such as peptic ulcer disease treatment. Peer influence accounted for kaolin consumption in 20 (11.3%). Fifty-two (29.4%) consumed kaolin due to cravings and the desire to take clayey materials. Those who consume clay to treat their morning sickness (nausea) were 28 (15.8%) and 53 (29.9%) consume clay for fun or just for its experience.

**Figure 2:**
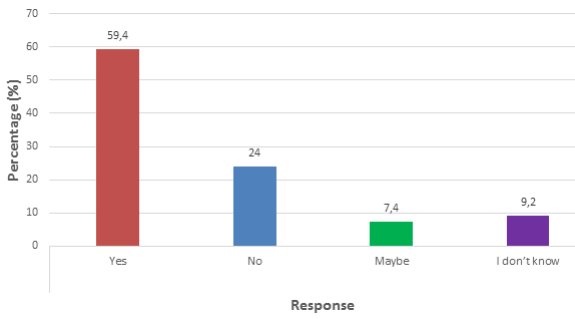
Some views of pregnant women on geophagy

**Figure 3:**
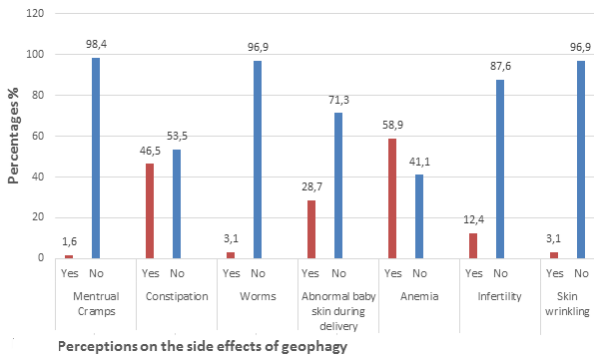
Pregnant women perceptions on the side effects of geophagy

**Pregnant women perceptions on the side effects of geophagy:** among 217 pregnant women interviewed on the side effects of geophagy, most, 129 (59.4%) said they knew there was some associated side effects with clay consumption (Figure 2). A sizeable proportion 52 (24%) said there was no side effect. A minority 16 (7.4%) said consuming clay might have some side effects and 20 (9.2%) said they do not know the effects of clay on the human body. One hundred and twenty-nine pregnant women who said clay had negative side effects gave some side effects they knew (Figure 3). A considerable proportion 76 (58.9%) said geophagy could cause anaemia, while 60 (46.5%) said it caused constipation, 2 (1.6%) said it elevated menstrual cramps. Four (3.1%) said it caused parasitic (worms) infestation. Thirty-seven (28.7%) said taking clay change the skin of your child to white during delivery. Sixteen (12.4%) said geophagy caused infertility. Only 4 (3.1%) of pregnant women said geophagy caused skin wrinkling.

**Association between demographics and geophagy practices:** Table 3 shows the associations between socio-demographic characteristics and awareness of side effects of pregnant women in the study. The age group of participants was significantly associated with awareness of side effects (χ^2^ = 24.027; p = 0.008). Ethnicity of the pregnant women was significantly associated with awareness of side effects (χ^2^ = 52.536; p < 0.001). Religion of participants was also significantly associated (χ^2^ = 18.138; p = 0.001). The occupation of pregnant women was equally associated (χ^2^ = 64.416; p < 0.001). The number of children pregnant women had was significantly associated (χ^2^ = 11.523; p = 0.021). Total monthly income of pregnant women was significantly associated (χ^2^ = 36.897; p < 0.001). Educational level had a significant association (χ^2^ = 31.049; p < 0.001) and the locality of these pregnant women also had significant association (χ^2^ = 19.754; p < 0.001). However, there was no significant association between household size and awareness of side effects (χ^2^ = 0.624; p = 0.732).

**Table 3:** Association between socio-demographic characteristics and awareness of side effects

Socio-demographic variable	Awareness of side effects			Total	Chi-square (p-value)
	Yes Freq. (%)	No Freq. (%)	Maybe Freq. (%)		
**Age group**					**24.027(0.008)**
16-19	11(8.5)	1(1.9)	1(6.3)	13(6.6)	
20-24	26(20.2)	9(17.3)	8(50.0)	43(21.8)	
25-29	4333.3%	11(21.2%)	2(12.5)	56(28.4)	
30-34	25(19.4)	10(19.2)	0(0.0)	35(17.8)	
35-39	19(14.7)	14(26.9)	3(18.8)	36(18.3)	
40-44	5(3.9)	7(13.5)	2(12.5)	14(7.1)	
**Ethnicity**					**52.536(<0.001)**
Ewe	117(90.7)	48(92.3)	12(75.0)	177(89.8)	
Akan	4(3.1)	4(7.7)	0(0.0)	8(4.1)	
Ga-Adangbe	8(6.2)	0(0.0%)	0(0.0)	8(4.1)	
Northerner	0(0.0)	0(0.0)	4(25.0)	4(2.0)	
**Religion**					**18.138(0.001)**
Christian	121(93.8%)	44(84.6%)	12(75.0%)	177(89.8%)	
Muslim	8(6.2%)	4(7.7%)	4(25.0%)	16(8.1%)	
Traditionalist	0(0.0%)	4(7.7%)	0(0.0%)	4(2.0%)	
**Occupation**					**64.416(<0.001)**
Self-employed	29(22.5%)	16(30.8%)	4(25.0%)	49(24.9%)	
Professional	44(34.1%)	8(15.4%)	0(0.0%)	52(26.4%)	
Office worker	12(9.3%)	8(15.4%)	0(0.0%)	20(10.2%)	
Trading	32(24.8%)	0(0.0%)	4(25.0%)	36(18.3%)	
Student	8(6.2%)	8(15.4%)	0(0.0%)	16(8.1%)	
Unemployed	43.(1%)	12(23.1%)	8(50.0%)	24(12.2%)	
**Number of children**					**11.523(0.021)**
	33(40.7%)	20(55.6%)	8(50.0%)	61(45.9%)	
	44(54.3%)	12(33.3%)	4(25.0%)	60(45.1%)	
	4(4.9%)	4(11.1%)	4(25.0%)	12(9.0%)	
**Household size**					**0.624(0.732)**
<6	92(71.3%)	40(76.9%)	12(75.0%)	144(73.1%)	
6 and above	37(28.7%)	12(23.1%)	4(25.0%	53(26.9%)	
**Total monthly income**					**36.897(<0.001)**
1000-1999	16(12.4%)	0(0.0%)	0(0.0%)	16(8.1%)	
800-999	28(21.7%)	12(23.1%)	0(0.0%)	40(20.3%)	
500-799	53(41.1%)	12(23.1%)	4(25.0%)	69(35.0%)	
100-499	28(21.7%)	24(46.2%)	8(50.0%)	60(30.5%)	
<100	4(3.1%)	4(7.7%)	4(25.0%)	12(6.1%)	
**Education Level**					**31.049(<0.001)**
None	4(3.1%)	0(0.0%)	0(0.0%)	4(2.0%)	
Primary	8(6.2%)	0(0.0%)	4(25.0%	12(6.1%)	
JHS/Middle school	45(34.9%)	28(53.8%)	12(75.0%)	85(43.1%)	
SHS/0’level	24(18.6%)	8(15.4%)	0(0.0%)	32(16.2%)	
Tertiary	48(37.2%)	16(30.8%)	0(0.0%)	64(32.5%)	
**Locality**					**19.754(<0.001)**
Urban	117(90.7%)	40(76.9%)	8(50.0%)	165(83.8%)	
**Rural**	**12(9.3%)**	**12(23.1%)**	**8(50.0%)**	**32(16.2%)**	

**Metal levels in clay samples:** the mean concentrations of essential metals were 1.38±1.5, 2.40±1.5, 7.74±1.5, 4.01±1.0, 13.24±2.2, 13.76±2.1 mg/Kg for Iron (Fe), Copper (Cu), Zinc (Zn), Potassium (K), Magnesium (Mg) and Sodium (Na) respectively while toxic metals concentration recorded were 1.63±0.03μg/kg, 4.72±0.9, 0.53±0.02, 1.85±0.3 mg/Kg for Arsenic (As), Manganese (Mn), Lead (Pb) and Nickel (Ni) respectively have been presented in Table 4.

**Table 4:** Mineral (macro and micro) levels in clay samples

Mineral Element	Mean concentration	Recommended Dietary Intake (RDI), WHO	
**Macro**			
Iron	1.38 ± 1.5 mg/Kg	18 mg	
Copper	2.40 ± 1.5 mg/Kg	0.9 mg	
Zinc	7.74 ± 1.5 mg/Kg	11 mg	
Potassium	4.01 ± 1.0 mg/Kg	3100-3500 mg	
Magnesium	13.24 ± 2.2 mg/Kg	280-350 mg	
Sodium	13.76 ± 2.1 mg/Kg	500-2400 mg	
**Micro**		WHO/FAO PMTDI (μg/Kg BW/day)	PMTDI for 60
Arsenic	1.63 ± 0.03 μg/Kg	3.0	180
Manganese	4.72 ± 0.8 mg/Kg	4.9 mg/Kg	294
Lead	0.53 ± 0.02 mg/Kg	3.0	180
Nickel	1.85 ± 0.3 mg/Kg	5.0	300

**Microbiological analyses:** predominantly, the species of bacteria which were common in all the samples were *Baccillus* and *Pseudomonas spp.* and their counts recorded ranged between 1.0x10^-1^-9.8x10^2^ and 4.0x10^1^-9.8x10^2^ CFU/g respectively. Likewise, *Mucor spp.* were predominant in samples not baked, collected from Anfoega and sold in retail shops (Anf/RET/NB) which recorded 2.0x10^1^ CFU/g. *Aspergillus spp.* were isolated from samples from Ho market which had been baked (Ho/mkt/B) (Table 5). Generally, there was an observed decrease in microbial counts as the ore was pretreated before sale for consumption.

**Table 5:** Micro-organisms isolated from the various samples plated on different media

Sample	Medium	TPC (CFU/g)	Microorganisms	
			Bacteria	Fungi
Anf/Ms/ORE	BA	9.8x10^2^	*Pseudomonas spp.*	
Anf/Ms/ORE	BA	9.8x10^2^	*Bacillus spp*.	
Anf/Ms/ORE	SDA	1.0x 10^1^	*Bacillus spp*.	
Anf/Ms/ORE	SDA		*Pseudomonas spp*.	
Anf/Ms/ORE	BA		*Pseudomonas spp*.	
Anf/Ms/ORE	PDA	9.5x10^2^	*Bacillus spp*.	
Anf/Ms/ORE	PDA		*Pseudomonas spp*.	
Anf/RET/NB	BA	2.0x10^1^	*Bacillus spp*.	
Anf/RET/NB	PDA	2.0x10^1^	*Bacillus spp*.	*Mucor spp*.
Anf/RET/NB	SDA	2.0x10^1^	*Bacillus spp*.	*Mucor spp*.
Ho/mkt/B	PDA	5.0x10^1^	*Pseudomonas spp.*	
Ho/mkt/B	PDA		*Bacillus spp.*	
Ho/mkt/B	SDA		*Bacillus spp.*	*Aspergillus spp*.
Ho/mkt/B	PDA		*Bacillus spp*.	
Ho/Mkt/NB	PDA	4.0x10^1^	*Pseudomonas spp*.	
Ho/Mkt/NB	SDA		*Bacillus spp.*	
Ho/Mkt/NB	BA	5.0x10^1^	*Bacillus spp.*	
Ho/Mkt/NB	BA		*Pseudomonas spp*.	
**Code**			**Interpretation**	
Ho/mkt/B			Ho market not baked	
Anf/RET/NB			Anfoega retail shop not baked	
Ho/Mkt/NB			Ho market not baked	
Anf/Ms/ORE			Anfoega mining site ore	

**Parasitological analysis:** no distinctive helminths, protozoans, cysts nor ova-like structures were identified. However, ova-like structures were seen but not confirmatory for routine parasites.

## Discussion

**Geophagy practices among pregnant women:** most noted and commonly reported key symptoms of pregnancy include nausea, vomiting, headache, dizziness and spitting [18]. Studies by [19] established that depression and its allied factors are very severe in the first trimester of pregnancy. The findings of [18] also pointed in this direction that pregnancy symptoms are most severe in the first trimester. It is however critical to note that the second and third trimesters are not free of pregnancy symptoms. In view of this, pica is practiced by pregnant women to curb these associated discomforts. Geophagy which is a common practice among pregnant women in Ghana and West Africa at large, is usually persistent. Data taken shows that most of these women heard of geophagy practices from either their friends or family or both. Most had consumed the clay once or several times in their lifetime. About half of them ate clay during childhood which indicates that geophagy is a practice that is built from an early age (childhood) before the woman reaches adulthood. This is in line with a research by [20] which suggests that the first geophagy experience is often during childhood due to influence of family members and friends or due to curiosity. Habitually, the consumption of clay ceases in adolescence, but is restarted during gestation. More than half of total participants who were interviewed had cravings to eat clay during pregnancy which support the claims made by [21] that women in their child bearing age have the desire to eat non-food items.

Results from our study also suggests a prevalence of 48.4% (n = 217) in the Ho municipality of Ghana which corroborates published findings of other researchers who reported prevalence of the same range in other parts of the country. In Ghana although information on pica is scanty, the few studies such as that conducted by [22] in Accra found 57% of her respondents practiced pica. Mensah *et al.* [23] reported a prevalence of 47.0% in Kumasi. Likewise, [18] also reported 52.12% among pregnant women in La-Nkwantanang municipality, Accra. However, relatively low prevalence of 28.49 % and 30.25% were reported by [24] (Accra) and [25] (Bibiani-Anhwiaso Bekwai, Western Region) respectively. Other documented surveys in some parts of Africa have also recorded high incidences of pica practices (especially geophagy) among pregnant women. Correspondingly, in urban and rural South African pregnant women, the prevalence rates were reported to be 38.3% and 44.0% [26]. In Nigeria, Sule and Madugu [27] reported a rate of 50% among pregnant women studied in Zaria. In Kenya, a light yellow soft stone (odowa) dug out from excavation sites at Kajulu hills in Kisimu District, is reported to be eaten by pregnant women [28]. On an interesting note, various types of clay are consumed by pregnant women in Malawi and further states that the practice is so common among the locals to the extent that almost every pregnant Malawian woman is expected to practice pica as a sign of pregnancy [29].

**Knowledge and perception about geophagy:** the results obtained also suggest smell and taste of clay, influenced the consumption by these pregnant women to a large extent and has been observed earlier by [30]. Changes in smell and/or taste for pica substances from the onset of pregnancy till delivery has been observed by pregnant women in this category. Thus, physiological changes may play a key role in pica development as suggested by [31]. A related study [32], also observed that people with heightened olfactory sensitivity during pregnancy had substantially more cravings than women with no olfactory changes. Damp earth was an important trigger for geophagy; some women reported cravings triggered by the smell of wet earth after it rained [33]. The physiological theory about clay or non-food item consumption is that eating clay or dirt helps relieve nausea, control diarrhoea, increase salivation, remove toxins and alter odor or taste during pregnancy [34]. Most pregnant women in order to relieve the physiological changes within their body during pregnancy take non-food items.

**Pregnant women perceptions on the side effects of geophagy:** strikingly, majority of the respondents had no knowledge on the adverse health implications clay consumption has on the human body. Most of the common perceptions given by these pregnant women on the side effect of geophagy are; it causes anaemia, constipation and affects the unborn child's skin colour making it appear pale. Although these are just perceptions, there have been proven associations between taking clay and anaemia as well as constipation [35] and it goes in line with claims made by Cornelius Celsus, Roman encyclopaedist who iterated that “people whose colour is bad when they are not jaundice are either sufferers of pain in the head or earth eaters” furthermore, a statement by Hippocrates of Kos (father of medicine) reveals so which reads “if a pregnant woman has the desire to eat earth or charcoal, and then eats them, the child born thereafter, would show signs of these things”.

**Mineral elements contained in the clay sample:** this study provides first-hand information on the elemental minerals of clay ores mined in Anfoega in the Volta region of Ghana. From the results, the levels of Arsenic obtained was below the mean exposure level of 3.0 μg/Kg BW/day set by the Joint FAO/WHO Expert Committee on Food Additives [36]. Results obtained in this study was slightly higher than findings of [37] who reported 0.0 (nil) mg/Kg of Arsenic in clay samples in Accra (Ghana). However, [38] reported a range of 218-271 ppm from clay samples in different parts of Kumasi Metropolis. Furthermore [39], also reported Arsenic levels of range 2.7-22.74 μg/g in nine (9) samples of clay samples collected from both Greater Accra and Kumasi of Ghana. Ingestion of large amounts of Arsenic can result in gastrointestinal symptoms such as vomiting, obstruction in the circulatory and nervous systems and ultimately death [40]. Lead concentrations were also below the mean exposure levels. This was in contrast with findings reported by [38] with a range of 549-622.92 μg/Kg. In line with our results [37], reported 2.36 ± 0.08 mg/100g. Lead consumption can result in adverse health effects such as the dysfunction of some vital organs such as kidneys, liver and heart. Mahurpawa [37] reiterated that multiple organs in the body are targeted by Lead due to systemic toxicity. Manganese and Nickel levels were also below the set limits. In Tanzania, Nyanza *et al.* [41] reported ranges of 2.3-128 and 2.9-1400 mg/Kg for Nickel and Manganese respectively. Nonetheless, [39] did not detect any Manganese in the clay samples from Ghana. Manganese is an essential nutrient involved in bone formation and carbohydrate metabolism. Information on the availability of these minerals in edible clay samples is scanty. The clay samples may contribute to dietary Ca, Fe, K, Na, and Zn but not Magnesium and Manganese. Na/K ratio in the body helps in controlling high blood pressure [42]. Umudi [43] reported ranges of 2.00 ± 0.00- 3.7±0.01 mg/100g, 20.65 ± 0.01-43.01±0.02 mg/100g and 63.01±0.01 mg/100g for Sodium (Na), Potassium (K) and Magnesium (Mg) respectively. Doe *et al* [39] also reported ranges of 28684-31941 μg/g for Potassium, 523-809 μg/g for Sodium, 0-22530 μg/g for Magnesium.

**Microbial analysis:** the bacterial analyses show that ore from Anfoega contained *Pseudomonas spp.* and *Bacillus spp.* the retails contained *Bacillus spp.* and *Mucor spp.* unbaked clay from the market contained *Aspergillus spp. Pseudomonas spp*. and *Bacillus spp.* whilst baked ones bought from Ho Asigame Market contained *Pseudomonas spp*. and *Bacillus spp.* The various media gave different microbial growth and results. The 3 media supported *Bacillus* and *Pseudomonas* species whilst SDA and PDA showed additional growth of fungi. However, the number of cells recorded differed in CFU/g; the least and highest being 2.0x10^1^ and 9.8x10^2^ respectively. The bacteria identified are of public health importance. Some *Bacillus spp.* are harmful to both humans, animals and plants although other *Bacillus spp*. are also beneficial to human. *Bacillus cereus* sometimes causes spoilage in canned foods and food poisoning of short duration (https://www.britannica.com/science/bacillus-bacteria). Due to the ubiquity of *Bacillus* species, they can mimic *Listeriosis* during pregnancy in which presentation of symptoms could range from transient bacteremia to serious systemic infection [44]. Some of the infections such as pneumonia, pan-ophthalmitis, visceral abscess, or musculoskeletal infections are caused by *Bacillus spp.* [45]. *Pseudomonas spp.* which were among the organisms identified also have pathogenic effects on humans. It can cause death among people with cystic fibrosis and immunocompromised people [46]. In Ghana, numerous pathogens and fecal coliforms were isolated from clay samples collected from different locations. Some of these fecal coliforms were *Staphylococcus spp, Klebsiella, Escherichia*, and *Shigella* and *Enterobacter spp.* [8]. Microbiological quality is affected since both sellers and buyers may affect the microbiological quality. Mining sites of clay can be contaminated with fecal matter. Tano-Debrah & Bruce-Baiden, [14] isolated coliform bacteria, *Staphylococcus* species, and yeasts from the external surfaces of white clay in Ghana. The predominant bacterial species that were found in sampled clay in Nigeria were *Bacillus subtilis*, Staphylococcus aureus, *Escherichia coli* and *Klebsiella* [47]. There were two helminths that were found in the cultured clay which were lumbricoides and hookworm [47]. There is the likelihood of contamination of processed clays by *Enterobacteriaceae, Staphyloccocus spp*. and other spore-forming pathogenic species. A range of 5000 to 7000 species of bacteria per gram of natural soil was reported by [48] and [49].

Fungi have less effects on human but they can produce toxins which may be harmful to the host. Pathogenic *Aspergillus spp.* produce mycotoxins. Aflatoxin, the most well-known and well-investigated mycotoxin, is known to carry the most potent carcinogenic activity as a natural product. It also carries acute toxicity to various human cells [50]. *Mucor spp.* that was identified, is a common fungus which is found in the soil. *Mucor spp.* cannot survive at 37°C but people who are immunocompromised can get serious health complications from this fungus [51]. The opportunistic infection by *Mucor spp.* is Zygomycosis which is an infection in mucous membranes, nasal passages and sinuses, eyes, lungs, skin, and brain, as well as renal and pulmonary infections and septic arthritis [52]. Although Toxocariasis and Ascariasis caused by the worms *Toxocara canis* and *Ascaric lumbricoides* respectively are known to be the most common parasitic infection associated with geophagy [53], there were no parasites (helminths etc.) detected in the investigated samples of the present study and this could be attributed to variation in efficiencies of methods employed in this study compared to other previously done studies [17].

## Conclusion

Geophagy is a common practice among pregnant women living in the Ho municipality in the Volta region, Ghana. Most of them consume it because of the smell and others for the taste. The physiological state of pregnancy also causes many to use it to treat nausea, other accompanying discomfort and in some instances to quench their hunger. The elemental analyses showed both beneficial and harmful minerals in the clay. Although there are beneficial minerals, accumulated effects of these heavy metals can lead to various complications in pregnancy, and the clay contains pathogenic organisms. These pathogens have a whole range of deleterious effects on the human body ranging from gastrointestinal infections to cancer.

### What is known about this topic

- Pica practice is prevalent in Africa;
- Geophagy is practiced in Greater Accra and Ashanti Regions Kumasi;
- Parasites are linked to clay consumption by pregnant women in Ghana.

### What this study adds

- Geophagy prevalence among pregnant women in Ho municipality of Ghana is 48.4%;
- Results of this research points out that clay consumed by pregnant women in Ho Municipality is free from parasites;
- Macro and micro elements investigated in the clay samples, were all within permissible levels recommended by World Health Organization (WHO).

## Competing interests

The authors declare no competing interests.
